# Modeling and Inverse Compensation of Cross-Coupling Hysteresis in Piezoceramics under Multi-Input

**DOI:** 10.3390/mi12010086

**Published:** 2021-01-15

**Authors:** Xiaochong Zhou, Lue Zhang, Zhan Yang, Lining Sun

**Affiliations:** Jiangsu Provincial Key Laboratory of Advanced Robotics, School of Mechanical and Electrical Engineering, Soochow University, Suzhou 215123, China; 20185229066@stu.suda.edu.cn (X.Z.); yangzhan@suda.edu.cn (Z.Y.); lnsun@hit.edu.cn (L.S.)

**Keywords:** piezoelectric actuator, hysteresis model, cross-coupling, inverse compensation

## Abstract

In the fast tool servo (FTS) system for microstructure surface cutting, the dynamic voltage hysteresis of piezoelectric actuators (PEAs) and the cutting force produced in the manufacturing affect the driving accuracy and the cutting performance. For a multi-input-single-output (MISO) cutting system, in this paper, a dynamic hysteresis model based on a rate-dependent Prandtl–Ishlinskii model is proposed. A backpropagation neural network (BPNN) is established to describe the cross-coupling effect between the applied voltage and external load. An inverse dynamic model is developed to compensate the nonlinearity of PEAs. The accuracy of the model and its inverse is discussed and the performance of the inverse feedforward compensator is validated through experiments.

## 1. Introduction

In recent years, components with microstructural surfaces have been widely used in optoelectronics, aerospace and other high-tech technologies. Ultra-precision manufacturing is necessary for these topological microstructural elements as the shape accuracy and the surface roughness requirement can be 10^−6^ m and 10^−9^ m [1,2], respectively. Due to the limitation of traditional cutting for a non-rotationally symmetrical microstructure, the fast tool servo (FTS) based on displacement characteristics of piezoelectric actuators (PEAs) was introduced. However, in the cutting process, there are significant differences between the displacements in the rising and descending period of the voltage, i.e., hysteresis nonlinearity. Moreover, the change of the driving frequency of PEAs can further lead to dynamic hysteresis and the cutting force can affect the driving accuracy of PEAs and the precision of the cutting as well. In order to satisfy the requirements in ultra-precision cutting, it is necessary to model and control the nonlinear characteristics.

For the hysteresis characteristics of piezoceramics, various hysteresis models have been proposed [3,4,5,6]. As most of these classical models are static, researchers have made a lot of improvements on the operators. Mayergoyz et al. proposed a generalized nonlinear Preisach model by introducing the integral term [7]. Compared with a traditional Preisach model, this model had better consistency attributes and could describe both first-order and second-order transition curves. Hu et al. focused on the influence of load on the accuracy of the traditional Preisach model and proved the influence of load and input signal frequency on the hysteresis characteristics [8]. Li and his team proposed and improved a variety of models based on a fuzzy system for hysteresis and the control of piezoelectric actuators and have made remarkable achievements in the parameter identification algorithm and feedforward control [9,10]. Gan et al. proposed a modified Duhem model to describe the rate-dependent hysteresis behaviors at high-frequency and high-amplitude excitations by combining trigonometric functions and derivatives of input signals based on the classical Duhem model [11]. Zhang et al. designed a dynamic operator by improving a Prandtl–Ishlinskii(PI) model operator, making the new model closely related to the input rate so that it had the ability to characterize the hysteresis dynamic characteristics [12]. Rakotondrabe proposed a multi-variable modeling method based on a rate-independent PI model. A feedforward compensator was also suggested based on the inverse multiplicative structure [13]. Chen et al. analyzed the voltage-force curve of the piezoelectric actuator and discovered that, similar to the hysteresis curve, the voltage-force curve possessed memory characteristics and rate-dependence [14]. Dong et al. verified the cross-coupling characteristics of piezoceramics under the combined action of voltage and external force by experiments and proposed a new model based on the traditional Preisach model to describe the hysteresis [15]. Ma et al. developed a generalized Preisach operator to estimate the dynamic coupling hysteresis under load and voltage [16].

This research on hysteresis modeling has improved the dynamic response characteristics of PEAs. Nevertheless, to control an FTS turning system, the electromechanical coupling characteristics of PEAs should be taken into consideration with the voltage excitation and the existence of the cutting force. In this paper, a dynamic cross-coupling hysteresis model with two inputs (voltage and load) is presented based on the classical PI model and an inverse feedforward compensator is also designed. The changing rate of the excitation signals is taken into account and the weights are replaced with dynamic ones. The cross-coupling effect of the two inputs is analyzed as well as the hysteresis effect under a voltage excitation. The experimental results show the accuracy of the model and its inverse.

## 2. Materials and Methods

### 2.1. Experimental Platform

**Figure 1:**
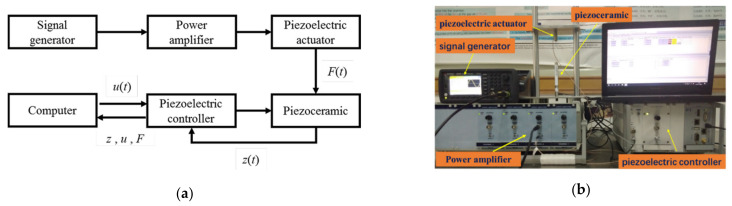
(**a**) Flow chart of piezoceramic performance, (**b**) experimental platform.

A PEA-based experimental platform was built. In order to verify the correctness of the model, the experiment was designed according to the block diagram below:

As is shown in Figure 1b, the voltage signal generated by the signal generator (33500B, Keysight, CA, USA), amplified by the power amplifier (E00. A4, Core Tomorrow, Harbin, China), was applied to the piezoelectric actuator (Pst150/7/20 VS12, Core Tomorrow, Harbin, China). The piezoelectric actuator provided the piezoceramic (P-841.6, Physik Instrumente, Karlsruhe, Germany) with a dynamic force, which could be calculated by the following formula:

(1) $$\begin{array}{c} F_{PZT}=\left(x_{l}-x_{a}\right)k \end{array}$$

where *x_l_* refers to the theoretical displacement of the piezoelectric actuator, *x_a_* denotes the actual displacement and *k* is the stiffness coefficient of piezoceramic. The parameters of the piezoelectric actuator are listed in Table 1.

The output displacement of the piezoceramic under the voltage excited by the piezoelectric controller (E-501.00N, Physik Instrumente, Karlsruhe, Germany) and the external load was collected and analyzed by the software (PIMikroMove, Physik Instrumente, Karlsruhe, Germany) matching with the piezoelectric controller in the PC. The feedforward compensator was designed based on the inverse model of the cross-coupling rate-dependent Prandtl–Ishlinskii (CRPI) model proposed and implemented in MATLAB/SIMULINK (Version R2018a, MathWorks). The compensated signal was applied to the piezoceramic by the piezoelectric controller, which communicated with MATLAB.

### 2.2. Cross-Coupling Hysteresis Prandtl–Ishlinskii Model and Its Inverse

A multi-input-single-output (MISO) CRPI model and its inverse were proposed to describe and to compensate the cross-coupling dynamic coupling hysteresis nonlinearity with the following steps. Firstly, a model was established to describe the hysteresis characteristics. Secondly, parameters, such as thresholds and weights, were identified by the least square method. Finally, the inverse model, including its form and parameters, was constructed to compensate the hysteresis.

#### 2.2.1. Cross-Coupling Hysteresis Prandtl–Ishlinskii Model

**Figure 2:**
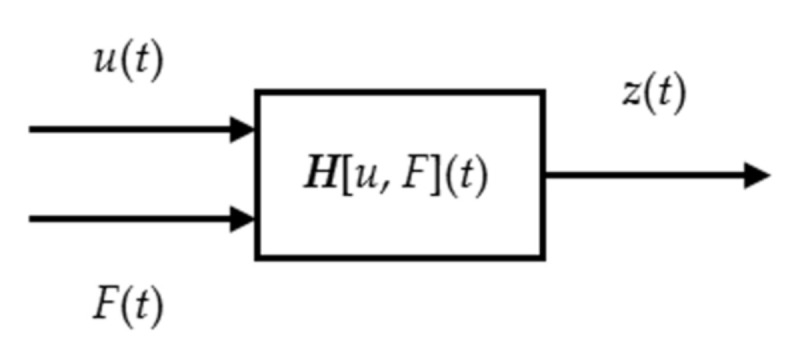
The hysteresis characteristic of the piezoceramic.

In the FTS turning system, the hysteretic characteristic of the PEA was presented in two variables: applied voltage *u* (*t*) and external load *F* (*t*). To describe the displacement characteristic, we introduced a function *H* [·] (Figure 2), which set up a relation between *u* (*t*), *F* (*t*) and the output displacement *z* (*t*) of the PEA [14].

As shown above, the output *H* [·] with both *u* (*t*) and *F* (*t*), the output *H_1_* [·] with *u* (*t*) and without *F* (*t*) and the output *H_2_* [·] with *F* (*t*) and without *u* (*t*) could be obtained respectively in the following formulation:

(2) $$z\left(t\right)=H\left[u,F\right]$$

(3) zt=H1ut for Ft=0 zt=H2Ft for ut=0.

The operator of the PI model was chosen as *H* [·] and the basic component of the PI-based hysteresis model was the backlash operator in the following formulation:

(4) $$y\left(t\right)=max\{u\left(t\right)-r,min[u\left(t\right)+r,y\left(t-T\right)]\}$$

where *r* was the threshold value, *w* was the weight value, *T* was the sampling period and *y* (*0*) = *max* {*u* (*0*) − *r*, *min* [*u* (*0*) + *r*, *0*]}. Different thresholds and weights were compounded to obtain the displacement:

(5) $$z\left(t\right)=\overset{n}{\underset{i=0}{\sum }}w^{i}\times max\{u\left(t\right)-r^{i},min[u\left(t\right)+r^{i},y\left(u,t-T\right).$$

Considering that the hysteresis of the piezoceramic is also affected by its dynamic characteristic, the dynamic rate of change of the input signal was introduced into the static weight of the classical PI model to form a rate-dependent hysteresis model as follows [17]:

(6) $$z_{1}\left(t\right)=H_{1}\left[u\right]\left(t\right)=\overset{n}{\underset{i=0}{\sum }}w_{i1}\times y\left(u,t\right)=\overset{n}{\underset{i=0}{\sum }}\left(k_{i}\times \dot{u}\left(t\right)+a\right)\times y\left(u,t\right)$$

(7) $$z_{2}\left(t\right)=H_{2}\left[u\right]\left(t\right)=\overset{n}{\underset{i=0}{\sum }}w_{i2}\times y\left(F,t\right)=\overset{n}{\underset{i=0}{\sum }}\left(m_{i}\times \dot{F}\left(t\right)+c\right)\times y\left(F,t\right).$$

**Figure 3:**
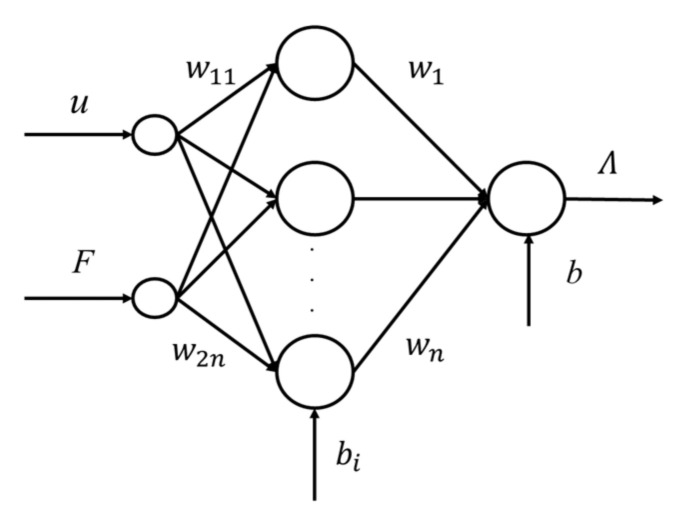
Neural Network diagram of *u* (*t*) coupling with *F* (*t*).

Equations (6) and (7) represented the voltage hysteresis and load hysteresis in piezoceramics, respectively, where *k* was the slope of ***H_1_***, *m* was the slope of ***H_2_*** and *a* and *c* referred to the offset coefficient of the dynamic weight. The cross-coupling effect between the two excitations was represented by **λ** [*u*, *F*] (*t*) and analyzed by a backpropagation neural network (BPNN) [18]. For *u* (*t*) and *F* (*t*), a BPNN was established in Figure 3.

As the relationship between *u*(*t*), *F*(*t*) and *z*(*t*) was nonlinear and the output of the network was limited to a small range, the sigmoid function was selected as the transfer function. The coupled displacement could then be expressed as:

(8) $$\lambda \left(u,F,t\right)=g\left\{\left[\sum_{j=1}^{n}w_{j}f\left(w_{1i}u+w_{2i}F+b_{i}\right)\right]+b\right\}$$

where *w_1i_* (*i* = 1, 2…n) and *b_j_* were the weights and the thresholds from the input layer to the hidden layer, respectively, *w_j_* and *b* were the weights and the thresholds from the hidden layer to the second layer, respectively, and *g*(*x*) was the transfer function.

Synthesizing Equations (6)−(8), a CRPI model was obtained:

(9) $$z\left(t\right)=H\left[u,F\right]\left(t\right)=H_{1}\left[u\right]\left(t\right)+H_{2}\left[F\right]\left(t\right)+\lambda \left[u,F\right]\left(t\right).$$

#### 2.2.2. Parameter Identification

Parameters that needed to be identified included *r*, *k* and *a* in the hysteresis operators. The parameters *w* and *b* in the BPNN were also required to be adjusted. For the piezoelectric actuators driven with a low-frequency, the hysteresis loop could be approximately rate-independent. In this case, a group of measured data under a low-frequency sinusoidal signal was used in Equation (5). Comparing the predicted output *z* (*t*) with the actual output *z_a_* (*t*), the error function was obtained as follows:

(10) $$E\left[z,z_{a}\right]\left(w,r,t\right)=z\left(t\right)-z_{a}\left(t\right)=\overset{n}{\underset{i=0}{\sum }}w^{i}\times y\left(t\right)-z_{a}\left(t\right).$$

By minimizing $E\left[z,z_{a}\right]\left(w,r,t\right)$, *w* and *r* in Equation (5) could be identified. The dynamic weights in Equation (6) could be similarly identified by minimizing the error function.

(11) $$E\left[z,z_{a}\right]\left(w,r,t\right)=z\left(t\right)-z_{a}\left(t\right)=\overset{n}{\underset{i=0}{\sum }}\left(k_{i}\times \dot{u}\left(t\right)+a\right)\times y\left(u,t\right)-z_{a}\left(t\right).$$

After obtaining the parameters in the rate-dependent PI model, the network should be trained by input and output samples. The *w* and *b* of the network were modified according to the steepest descent backpropagation (SDBP) algorithm [19]. Let *k* be the number of iterations and the revision of each layer was carried out as follows:

(12) $$x\left(k+1\right)=x\left(k\right)-\alpha g\left(k\right).$$

In the formula, *x* (*k*) was the connecting weight vector or threshold vector between layers in the *k*th iteration, $g\left(k\right)=\partial E\left(k\right)/\partial x\left(k\right)$ was the gradient of the output error of the neural network to the weight value or the threshold value, *α* was the learning rate and its default value was 0.01 and $E\left(k\right)=\frac{1}{n}\sum_{i=1}^{n}{\left(y_{i}-\Lambda_{i}\left(k\right)\right)}^{2}$ was the total error performance function of the network output of the *k*th iteration.

#### 2.2.3. Inverse Feedforward Compensation

Inverse model compensation is the most common method in feedforward control and can realize the approximate linearization by connecting a hysteresis inverse model in front of the piezoceramic. The specific process was as follows. According to the required displacement, a voltage *u*_1_(*t*) could be obtained by the inverse model and the *u*_1_ (*t*) was then applied to the piezoceramic to observe the displacement. The output of the CRPI cascaded with its inverse model was as follows.

(13) $$z\left(t\right)=H\left[H^{-1}\left(\hat{z}\right)\right]t$$

where $\hat{z}\left(t\right)$ is the desired displacement. In order to obtain the inverse hysteresis model, the analytical inversion method rather than the direct inverse model method was employed as the CRPI model had been identified in the previous work of this project. Based on the idea of the analytical method, the inverse model could be expressed as:

(14) $$u\left(t\right)=H^{-1}\left[u,F\right]\left(t\right)=H_{1}^{-1}\left[z_{1}\right]\left(t\right)=\overset{n}{\underset{i=0}{\sum }}w_{i1}^{\prime }\times z\left(t\right)$$

(15) $$F\left(t\right)=H^{-1}\left[u,F\right]\left(t\right)=H_{2}^{-1}\left[z_{2}\right]\left(t\right)=\overset{n}{\underset{i=0}{\sum }}w_{i2}^{\prime }\times z\left(t\right).$$

The relationship between the threshold and weight coefficient in the PI model and its inverse could be deduced according to the slope and the junction point as follows [20,21].

(16) $$r_{i}^{\prime }=\overset{i}{\underset{j=0}{\sum }}w_{j}\left(r_{i}-r_{j}\right)$$

(17) $$w_{0}^{\prime }=\frac{1}{w_{0}}$$

(18) $$w_{i}^{\prime }=\frac{-w_{i}}{\left(\sum_{j=0}^{i}w_{j}\right)\left(\sum_{j=0}^{i-1}w_{j}\right)}.$$

The parameters in the inverse of the rate-dependent PI model could be obtained by replacing *w* with the dynamic weight according to Equations (16)–(18). The specific expressions were as follow.

(19) $$r_{i}^{\prime }=\overset{i}{\underset{j=0}{\sum }}w_{j}\left[\dot{u}\left(t\right)\right]\left(r_{i}-r_{j}\right)$$

(20) $$w_{0}^{\prime }\left[\dot{u}\left(t\right)\right]=\frac{1}{w_{0}\left[\dot{u}\left(t\right)\right]}$$

(21) $$w_{i}^{\prime }\left[\dot{u}\left(t\right)\right]=\frac{-w_{i}\left[\dot{u}\left(t\right)\right]}{\left(\sum_{j=0}^{i}w_{j}\left[\dot{u}\left(t\right)\right]\right)\left(\sum_{j=0}^{i-1}w_{j}\left[\dot{u}\left(t\right)\right]\right)}.$$

## 3. Results

**Figure 4:**
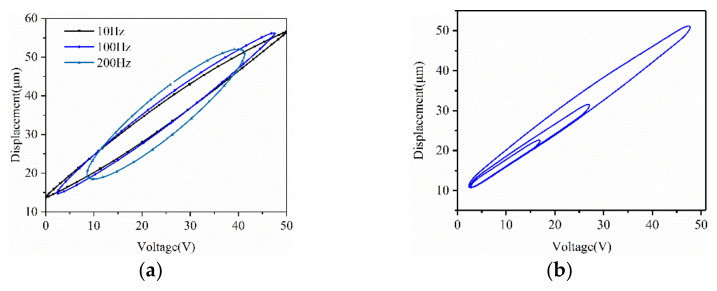
(**a**) Influence of the frequency of *u* (*t*) on the hysteresis, (**b**) Multi-hysteresis loops under voltage.

Two groups of experiments (Case 1: *u* (*t*) = *Asin2πf*), *F* (*t*) = 0; Case 2: *u* (*t*) = *Asin2πf*, *F* (*t*) = *Bsin2πf*) were carried out on the platform to validate the dynamic characteristics of piezoceramics. Figure 4a shows the relationship between the exciting voltage $u\left(t\right)=50sin\left(2\pi ft\right)\;\left(0s\ll t\ll \frac{T}{2}\right)$ (*f* = 10 Hz, 100 Hz, 200 Hz) and the displacement. Figure 4b is the multi-hysteresis loops under voltage input *u* (*t*) = 50*sin*10*πt*, *t* ϵ [0,0.1]; *u* (*t*) = 30*sin* [10*π* (*t*-0.1)], *t* ϵ [0.1,0.2] and *u* (*t*) = 20*sin* [1 0*π* (*t*-0.2)], *t* ϵ [0.2,0.3].

It can be seen from Figure 4 that in a hysteresis loop, the gap of the curve firstly becomes wider with the increasing of the voltage in the first quarter period and then becomes narrower in the next quarter period. In the descending period of the voltage, the trend is similar. The maximum width reached 15μm. In hysteresis loops under voltage excitations with the same amplitude and different frequencies of 10 Hz, 100 Hz and 200 Hz, the maximum width of the loop was about 7μm, 9μm and 15μm, respectively, and the length was about 50 V, 42 V and 30 V, respectively. This showed a trend that the ellipse of the hysteresis got shorter and wider with a higher frequency. In multi-hysteresis loops under voltage excitations with the same frequency and different amplitudes of 50 V, 30 V and 20 V, the amplitude of the input influenced the width of hysteresis loop mainly by affecting the return distance in the hysteresis loop and the loop got wider with a larger amplitude.

**Figure 5:**
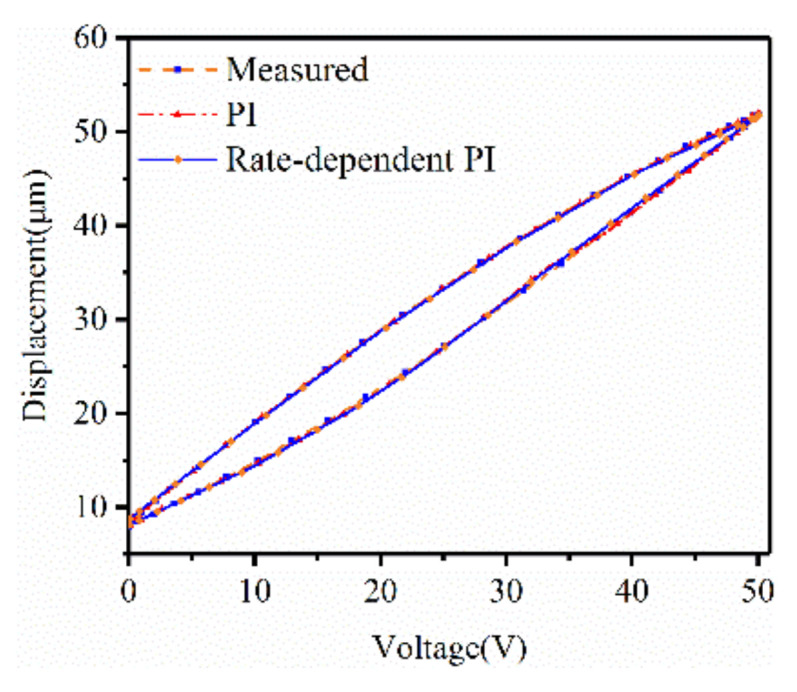
Hysteresis curve measured and hysteresis modeling based on classic a Prandtl–Ishlinskii (PI) method and a rate-dependent PI model.

The excitation $u\left(t\right)=50sin\left(5\pi t\right)\;\left(0s\ll t\ll 0.2s\right)$ was applied as the actuating signal to the system to identify the parameters in Equations (5) and (6). The classical PI model and rate-dependent PI model identified are shown in Figure 5 and the identified parameters are listed in Table 2.

According to Figure 5, the average errors of the classical PI model and rate-dependent PI model were 0.8173 μm and 0.3128 μm, respectively. The modelling error was reduced by about 60%, which showed that, compared with the classical PI model, the accuracy of the rate-dependent PI model proposed was improved.

For the identification of the model in Equation (7), an action of external $F\left(t\right)=60sin\left(5\pi t\right)\;\left(0s\ll t\ll 0.2s\right)$ was applied as the excitation to the system. In order to obtain λ, we took *u* (*t*) and *F* (*t*) as the two inputs of the BPNN and the difference between the displacement under the coupling action and the displacement under *u* (*t*) and *F* (*t*) separately as the output. The weights and thresholds of each layer and the number of hidden layer nodes were obtained by training the selecting data from the samples. The maximum training error, which was 2.2 nm, and the minimum error, which was 0.5 nm, between the training value and the actual value verified the feasibility of the network.

**Figure 6:**
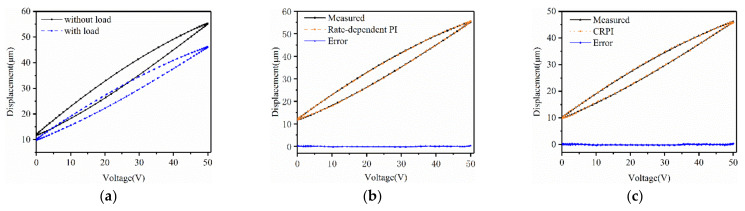
(**a**) Measured displacement with and without load; (**b**) Predicted and measured displacements of rate-dependent PI and errors; (**c**) Predicted and measured displacements of the cross-coupling rate-dependent Prandtl–Ishlinskii (CRPI) method and errors.

The measured displacements in Case 1 and Case 2 are shown in Figure 6a. For the experimental data in Case 1, we used the rate-dependent PI model with a single voltage excitation to fit. The identified rate-dependent hysteresis model is shown in Figure 6b. As for the displacements measured in Case 2, the CRPI model proposed was employed to approximate the curve. The output of the CRPI model and the prediction errors are shown in Figure 6c. The maximum error, minimum error and average error are listed in Table 3. It could be observed that the identified hysteresis model agreed with the measured hysteresis of the PEA.

**Figure 7:**
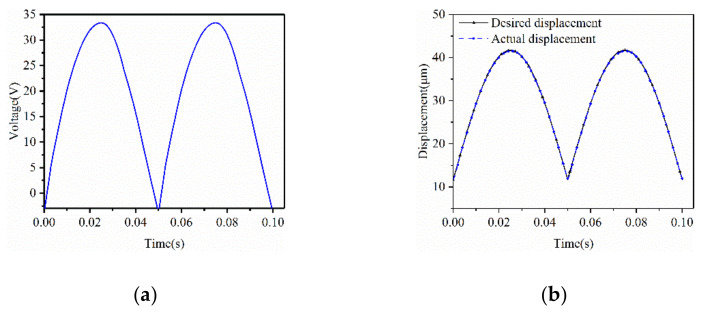
(**a**) The exciting *u* (*t*) after compensation of the rate-dependent PI model; (**b**) Actual displacements after compensation and desired displacements.

In the aspect of compensation, we first verified the inverse feedforward compensator by the experimental data in Case 1. The compensated voltage obtained according to Equation (14) is shown in Figure 7a. Figure 7b shows the compensated displacement of the model. It could be observed that the displacement measured after *u* (*t*) was compensated agreed with the required displacement. The average error was 0.3575 μm and the minimum error was 6.9 nm, which showed that the compensation was effective.

**Figure 8:**
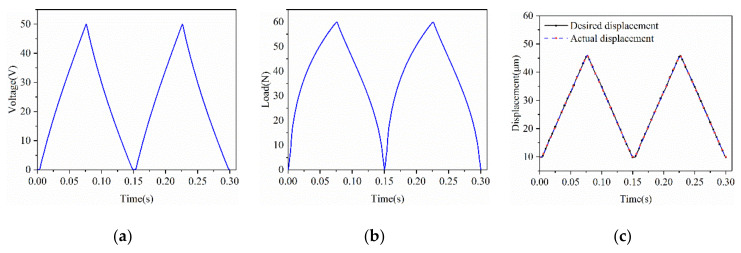
(**a**) The exciting *u* (*t*) after compensation of the CRPI model; (**b**) The exciting *F* (*t*) after compensation of the CRPI model; (**c**) Actual displacements of the CRPI model after compensation and desired displacements.

As for the desired displacements *z* (*t*) = 480 *t* + 10, *t* ϵ [0,0.075]; *z* (*t*) = 480 *t* + 82, *t* ϵ [0.075,0.15] of the PEA under both voltage and load excitation, the compensated voltage and the compensated load are shown in Figure 8a,b, respectively. The required displacements and the measured displacements after compensation are shown in Figure 8c. The maximum error and average error are 0.5284 μm and 0.1527 μm, respectively. When there is no inverse feedforward compensator applied, the maximum error and average error can be 3.4861 μm and 1.7485 μm, respectively. The hysteresis effect is reduced by about 91% and this proves that the feedforward control method proposed is significantly effective in compensating the hysteresis characteristic.

## 4. Discussion

Hysteresis modeling and compensation have become important issues in the micro-nano actuation of PEAs. In this paper, a dynamic cross-coupling model based on the classical PI model was proposed to describe the hysteresis of PEAs in FTS when they were subjected to exciting loads and voltage signals. The rate-dependent hysteresis characteristic was analyzed under a voltage excitation and a load excitation and a BPNN was presented to describe the dynamic cross-coupling effect. The parameters, including the threshold and weight of the backlash operators and the neural network, were identified. In terms of compensation, an inverse model feedforward compensator was designed. Both voltage compensation and load compensation were used and the parameters in the inverse model were also identified. A hysteresis experiment and a compensation experiment were carried out. It was experimentally shown that the model could provide good accuracy and the compensation method was effective.

However, there are still a few problems that need to be noticed. For example, the load applied to the piezoceramic was calculated by its stiffness and residual displacement and its accuracy needs to be improved. In the future, a better data processing method needs to be proposed. What is more, there is a certain distance between the effect of the inverse feedforward compensation and the required target so a feedback control method will be applied to further improve the performance of the PEAs.

## Figures and Tables

**Table 1:** Parameters of Pst150/7/20 VS12.

Parameters	Value
Open loop stroke (μm)	19
Stiffness (N/μm)	60

**Table 2:** Identified parameters.

No.	Classical PI		Rate-Dependent PI		
	*r*	*w*	*r*	*k*	*a*
1	0	0.644080	0	−0.001260	0.001178
2	5	0.124298	5	0.006158	3.218373
3	10	0.121281	10	0.012658	6.441789
4	15	0.089255	15	0.019138	9.661166
5	20	0.080102	20	0.025697	12.88137
6	25	−0.254660	25	0.032294	16.10247
7	45	0.543367	45	0.037195	19.32157

**Table 3:** Output errors.

	Rate-Dependent PI	CRPI
Maximum error (μm)	0.5342	0.4959
Minimum error (μm)	0.000016	0.000006
Average error (μm)	0.3128	0.2363

## Data Availability

The data presented in this study are available on request from the corresponding author. The data are not publicly available due to privacy.
